# Correlation of medical students' situational motivation and performance of non-technical skills during simulation-based emergency training

**DOI:** 10.1186/s12909-020-02247-6

**Published:** 2020-10-08

**Authors:** Leonie Schulte-Uentrop, Jonathan S. Cronje, Christian Zöllner, Jens C. Kubitz, Susanne Sehner, Parisa Moll-Khosrawi

**Affiliations:** 1 Department of Anesthesiology, University Medical Center Hamburg-Eppendorf, Martinistr. 52, 20246 Hamburg, Germany; 2 Department of Anesthesiology and Intensive Care Medicine, University Medical Center of the Paracelsus Medical Private University Nürnberg, Prof.-Ernst-Nathan-Str. 1, 90419 Nürnberg, Deutschland; 3 Department of Medical Biometry and Epidemiology, University Medical Center Hamburg-Eppendorf, Martinistr. 52, 20246 Hamburg, Germany

**Keywords:** Non-technical skills, Simulation-based medical education, Motivation

## Abstract

**Background:**

Non-technical skills (NTS) are an indispensable element of emergency care and need to be prevalent alongside with good technical skills. Though, questions of how to teach (instructional design) and improve NTS effectively remain unresolved. One adjustment screw to enhance performance of NTS, which is detached from instructional designs and learning efforts might be motivation. Theoretical models and observational studies suggest that high levels of intrinsic (situational) motivation result in better performance and better learning. Therefore, this study analyzed the influence of motivation on performance of NTS, by exploring if high levels of intrinsic motivation lead to better performance of NTS in medical students.

**Methods:**

In this prospective cross-sectional cohort study, the authors assessed the correlation of situational motivation and performance of NTS within a cohort of 449 undergraduates in their 1st to 4th year of medical studies, in a total of 101 emergency simulation trainings. Situational motivation was measured with the validated Situational Motivation Scale (SIMS), which was completed by every undergraduate directly before each simulation training. The NTS were evaluated with the Anesthesiology Students´ Non-Technical skills (AS-NTS) rating tool, a validated taxonomy, especially developed to rate NTS of undergraduates.

**Results:**

Student situational motivation was weakly correlated with their performance of NTS in simulation-based emergency trainings.

**Conclusion:**

Although motivation has been emphasized as a determining factor, enhancing performance in different fields and in medicine in particular, in our study, student situational motivation was independent from their performance of NTS in simulation-based emergency trainings (SBET).

## Background

High risk working places, like aviation or emergency-medicine, entail hazards for errors which can have disastrous consequences. Up to 80% of these adverse events are based on human factors [1].

To counter human errors in medicine, several training programs have been developed in the past – e.g. Anesthesia crisis resource management (ACRM), which emphasize cognitive and interpersonal skills, like communication, leadership, resource management and situational awareness [2]. These skills can be summarized as non-technical skills (NTS) [3, 4]. Over the past years, NTS have gained more attention and have been identified as inevitable for best patient care and safety [5, 6]. NTS are acquired through the socialization process of every human being and are heterogeneously distributed [7]. Despite the need to integrate teaching of NTS already in undergraduate medical education, current curricula usually do not address NTS in emergency training. Although some efforts were recently made to include teaching and assessment of NTS in undergraduate education, [8] it is not yet clear how this integration and which instructional designs might provide best learning outcomes.

Only few studies have investigated the effects of interventions like extensive debriefings or repeated exposures to simulation-based training, on performance and improvement of NTS [9, 10]. The questions of how NTS can be conveyed effectively and how performance of NTS can be improved remain unanswered. It is known that NTS are not necessarily promoted through clinical routine [11, 12] and therefore other adjustment screws than instructional designs should be identified.

Motivation presumably might be one determining factor in improving performance of NTS. That does not mean the motivation to learn NTS, as even high motivation to learn NTS might not result in better performance of NTS [13], because learning processes for achieving certain skills differ from classical factual learning (which can be mastered through diligence). Rather, the existing levels of individual motivation for engaging in activities could be an adjustment screw for enhancing performance of NTS. The positive impact of motivation on performance has been demonstrated in the past decades in several fields, e.g. psychology and education [14–17]. One of the leading motivation theories is the „Self Determination Theory “(SDT), described by Deci and Ryan [18], which has shown broad applicability and importance in medical education [13, 19, 20]. SDT postulates that humans have an innate will to grow, which can be supported or hampered by intrinsic or extrinsic factors or situations [21]. The growth is determined by the satisfaction of three basic psychological needs: autonomy, competency and relatedness [22]. In SDT, levels of motivation are described along a spectrum, with “intrinsic motivation” at one end and “amotivation” (having no motivation at all – when the person experiences a lack of competence or the reason for a task is not seen) at the other end [22]. Intrinsic motivation (the most autonomous form) is present, when activities are pursued for personal enjoyment, leading to inherent satisfaction. When external sources form the reason why an activity is carried out, extrinsic motivation is present. Extrinsic motivation has different levels of self-determination and autonomy: external, introjected, identified and integrated regulation [22]. The least autonomous regulation, *external regulated* behavior, is based on demands, punishment or possible rewards [23]. *Introjected regulation* is more autonomous than external, but the activity or rule is still seen as made by others and the activity is carried out to avoid guilt [24]. *Identified regulation* is more autonomous than *introjected regulation*. Here, the pursued activity is accepted as important and the person identifies itself with the task. The most autonomous regulation of extrinsic motivation is *integrated regulation*. Here, activities are connected to oneself [25]. All these motivations are present at a specific time, for a specific activity and therefore they are referred to as “situational motivation” [26].

Intrinsic, integrated and identified regulation are summarized as “autonomous self-regulation” and external and introjected regulation are summarized as „controlled self-regulation “ [24]. In medical students, high levels of autonomous regulation or intrinsic motivation lead to better well-being, better learning and academic achievement [27, 28].

But, to our best knowledge, the correlation of medical students´ motivation and their performance of non-technical skills (NTS) in medical emergency training has not been described.

The undergraduate anesthesiology curriculum (1st to 4th year of medical studies) is, among other teaching units, composed of different anesthesiology/emergency simulation trainings, which build upon each other.

These trainings are interactive and focus on technical and non-technical skills.

In this prospective, observational cohort simulation study, we investigated the correlation of students´ motivation and their performance of NTS during simulation-based emergency training (SBET) in four cohorts of medical students.

We hypothesized that high levels of autonomous motivation might lead to better performance of NTS.

## Methods

### Study design and participants

We performed this prospective cohort simulation study, at the Department of Anesthesiology of the University Medical Center Hamburg-Eppendorf, Germany, during the Winter semester 2018/19.

The undergraduate curriculum of the University Medical Center Hamburg- Eppendorf, is based on the spiral curriculum of Harden [29]. To foster learning, some teaching and learning goals are repetitively addressed and trained in consecutive semesters. This instructional design allows to reiterate and to extend the teaching contents and learning goals. Global learning objectives are composed of different lectures and training sessions of different disciplines, creating a learning module. Each module is assigned to its year of medical study (semester).

The undergraduate anesthesiology teaching units and trainings are designed since 2012, in the sense of the above mentioned learning spiral, to facilitate the retention of knowledge, through the constant repetition: Medical students of the University of Hamburg participate in their 1st to 4th year of medical studies in compulsory anesthesiology/emergency trainings, which build upon each other.

The 1st year students partake in one training session (Trauma training), the 2nd year students partake also in one training session (Advanced cardiac life support I), the 3rd year students in two training sessions (Advanced cardiac life support II and Operating room (OR) simulation) and the 4th year students partake in two training sessions (Advanced cardiac life support III (Respiratory emergencies, Cardiac emergencies)).

Each semester is divided into a lecture- and a lecture free period, followed by two examination weeks. The exams are scheduled after the lecture free period to provide enough time for each undergraduate to prepare for the semester exams.

The anesthesiology training sessions are all scheduled within the lecture period of the semester, in which all lectures and trainings of all assigned disciplines are conducted. No exams were scheduled near the time of the anesthesiology simulation trainings or other lectures.

Four hundred forty-nine students (*n* = 182 male, *n* = 267 female; 100%) in their 1st, 2nd, 3rd and 4th year of medical studies, who attended the compulsory anesthesiology/emergency simulation trainings, were included in the study. Motivation and NTS were assessed in a total of 101 trainings. A maximum of 17 students participated per training unit.

### Anesthesiology and emergency trainings

Every simulation training has a set of standardized simulation scenarios, which are comparable regarding technical skills (TS) and non-technical skills (NTS). With each subsequent semester, the scenarios become more complex.

High fidelity simulators (Resusci Anne Laerdal) are used, which are suitable for training technical skills such as endotracheal intubation, defibrillation or drug administration. In each training, the undergraduates are divided into groups of three, rotating through the pre-set simulation scenarios. One of the undergraduates takes the role of the leading anesthesiologist/emergency physician and the other two of anesthetic co-workers. Each simulation scenario is supervised by the course instructor (an anesthesiologist who is experienced in medical education). After each simulation scenario, a debriefing on TS and NTS takes place.

Situational motivation of all participating students was assessed prior to each training session with the Situation Motivation Scale (SIMS) questionnaire [30]. NTS were assessed for the student taking the team-leader (anesthesiologist/emergency physician) role, using the “Anesthesiology Students´ Non-Technical Skills” (AS-NTS) rating tool [31]. Table 1 gives an overview of the number of included AS-NTS and SIMS assessments.

**Table 1:** Number of included AS-NTS ratings and SIMS questionnaires assessed in the winter semester 2018/19 at the University Medical Center Hamburg Eppendorf

	Total	1st year	2nd year	3rd year	4th year
**SIMS questionnaires** ^**a**^	744	114	122	260	251
**NTS ratings with corresponding SIMS questionnaires** ^**b**^	422	80	60	113	169

*Abbreviation: AS-NTS* Anaesthesiology Students’Non-Technical skills, *SIMS* Situational Motivation Scale, *NTS* Non-technical skills

^a^ Data from 12 teaching units were incomplete and excluded from analysis

^b^A total of 422 AS-NTS ratings were completed. Only ratings of students who had filled out a complete corresponding SIMS questionnaire were analysed for the calculation of correlation

### Assessment

#### SIMS – situation motivation scale

A translated version (German) [32] of the Situation Motivation Scale (SIMS) [30], adapted by Gillet et al. [26], was filled out by every student at the beginning of each training session. The SIMS measures the different types of motivation for an activity at a specific point of time. It examines the important question why the individual shows a certain behavior and engagement [33].

The adapted version of the SIMS has five sub-scales, with four items per subscale, measuring intrinsic motivation, external-, identified-, introjected regulation and amotivation. Each item has a 7-point Likert scale (1 = “*Does not correspond at all*” and 7 = “*Corresponds exactly*”). Students were asked to specify the degree to which each item represents a reason for them to participate in the training. A computed autonomous motivation index was calculated by adding and averaging the intrinsic motivation and identified regulation. A controlled motivation index was computed parallel to that by adding and averaging external- and introjected regulation [26, 30]. Validity and reliability of the SIMS, as well as the adapted version, have been supported in several studies [34].

#### Anaesthesiology students` non-technical skills (AS-NTS)

NTS were assessed by the AS-NTS evaluation sheet (“Anaesthesiology Students` Non-Technical skills”) [31] which was filled in by the course instructor, who supervised the simulation scenario. All instructors were familiar with the AS-NTS, experienced in undergraduate medical education and working in anesthesiology and emergency medicine.

In AS-NTS, performance is rated on three dimensions. Each dimension (subscale has 5 items, which discriminate the performance on a five-point Likert scale (1 = “*very good”*; 5= “*very bad performance”*). An underlying skill structure is used to give behaviourally anchored rating examples (“*good*” or “*poor*”).

AS-NTS provides single scores for each dimension, as well as computed sum score (AS-NTS_SUM) by adding the dimensions.

The dimensions of the AS-NTS are:

- *Dimension 1: Planning tasks, prioritizing and conducting*
- *Dimension 2: Teamwork: exchanging information and leading the team*
- *Dimension 3: Team orientation*

Reliability and validity of AS-NTS have been previously supported [31].

### Statistical analysis

Statistical analysis was performed with SPSS (version 23.0, IBM Corp., Armonk, New York, USA). Descriptive statistics were used to describe the levels of reported motivational qualities. Mean differences in motivation and NTS were compared by year of medical school with analysis of variance (ANOVA). Significant differences between the different groups were analyzed by a follow-up post hoc test (Bonferroni correction). The Pearson correlation coefficient was used in order to test independence between motivation and performance of NTS.

Internal consistency of the adapted German SIMS version was analyzed, calculating Cronbach alpha for each sub-scale.

## Results

### Situational motivation

All students reported to be predominantly autonomously motivated with corresponding low levels of external regulation (Table 2). Comparing the year of their study, different year medical students reported varying qualities of situational motivation (Table 2). 2nd year students´ levels of identified motivation and levels of autonomous regulation were significantly higher than 3rd and 4th year students (*p <* .000). Furthermore, 1st year students were significantly higher on identified levels of motivation than 4th year students (*p* < .000) (Table 2).

**Table 2:** Means (standard deviation) and ANOVA results of different situational motivational and AS-NTS scores of different year undergraduates, assessed in the winter semester 2018/19 at the University Medical Center Hamburg Eppendorf

Situational Motivation	1st year	2nd year	3rd year	4th year	ANOVA			Crohnbach alpha
					F (df)	p	η^**2**^	
*Intrinsic*	5.87 (.81)	6.00 (.63)	5.85 (.76)	5.85 (.80)	1.23 (3)	.27	.005	.76
*Identified*	5.70 (.81)^B^	5.91 (.81)^A^	5.39 (1.13)^A^	5.36 (1.18)^AB^	9.67 (3)	.000*	.04	.68
*Introjected*	2.88 (1.17)	2.84 (1.28)	2.78 (1.27)	2.84 (1.33)	.23 (3)	.88	.001	.74
*Extrinsic*	1.89 (.90)	1.85 (1.02)	1.76 (.83)	1.82 (.88)	.66 (3)	.58	.003	.78
*Amotivation*	1.56 (.82)	1.42 (.59)	1.48 (.74)	1.43 (.68)	1.30 (3)	.27	.005	.75
*Motivational Indices*								
*Autonomous*	5.78(.70)	5.95(.61)^A^	5.62(.84)^A^	5.60 (.88)^A^	4.49 (3)	.000*	.003	
*Controlled*	2.38 (.86)	2.34 (1.04)	2.27 (.86)	2.33 (.91)	.53 (3)	.66	.002	
**AS-NTS**								
*Dim_1*	2.29(.83)	2.2 (1.01)	2.00(.86)	1.95(.81)	2.87 (4)	.023*	.02	
*Dim_2*	2.24(.83)	2.18(.90)	1.94(.90)	1.93(.83)	2.87 (4)	.023*	.02	
*Dim_3*	2.19(.80)	2.24 (1.04)	1.92(.89)	1.84(.78)	5.00 (4)	.001*	.04	
*Sum score*	6.73 (2.20)	6.62 (2.76)	5.86 (2.43)	5.69 (2.01)	4.50 (4)	.001*	.04	

Abbreviations: Dim = dimension

*p < 0.05 A Post-hoc analysis with Bonferroni-correction for multiple testing revealed that 2nd years´ levels of identified motivation and levels of autonomous regulation was significantly higher than 3rd and 4th year students (*p* < .000). B Furthermore, 1st year students were significantly higher on identified levels of motivation than 4th year students (*p* < .000). AS-NTS scores of 4th year students were significantly better than first year students on all three dimensions and on the sum score (*p* = .023; .023; .001; .001)

### Correlation of students´ situational motivation and NTS

Amotivation was weakly correlated to poor performance on dimension one (planning tasks, prioritizing and conducting, *p =* .04) and to the sum score of AS-NTS (*p =* .04). Furthermore, identified regulation (*p =* .01) and autonomous motivation (*p =* .03) showed a weak correlation to poor performance on dimension three of AS-NTS (Team orientation). These results were statistically significant but not relevant (Table 3) because the correlation was very weak (r below 0.3). Weak correlations might be statistically significant but they do not have any informational value [35].

**Table 3:** Correlation of NTS and SIMS scores assessed during simulation-based emergency training in the winter semester 2018/19 at the University Medical Center Hamburg Eppendorf

DIM1 ***r=***	Situational Motivation					Motivation Indices	
	Intrinsic .005	Identified .078	Introjected .048	Extrinsic .034	Amotivation .101^*^	Autonomous .054	Controlled .051
**DIM2** ***r=***	−.022	.026	.028	.031	.089	.009	.035
**DIM3** ***r=***	.047	.122*	.093	.045	.082	.104*	.088
**SUM** ***r=***	.011	.084	.063	.041	.101^*^	.062	.065

*Abbreviations*: *r* Pearson correlation Coefficient

*DIM* Dimension of the AS-NTS rating tool

*SUM* Sum of the AS-NTS rating tool score

*p < 0.05

The scores for all NTS dimensions, measured by the AS-NTS, showed a progress in NTS over the years (decrease in scoring = better performance, 1 = “*very good”*; 5= “*very bad performance”*). AS-NTS scores of 4th year students were significantly better than first year students on all three dimensions and on the sum score (Table 2).

## Discussion

In this study, high levels of autonomous situational motivation of students did not correlate with better performance of NTS in simulation-based emergency training.

The urgent need to focus early on NTS in medical education and training has been emphasized and therefore strategies to foster learning NTS should be identified [8]. We hypothesized, that next to instructional designs focusing on NTS, motivation might to be a determining factor that could enhance performance of NTS in undergraduates. Previous findings in the field of motivation showed that students with high motivation achieve higher academic degrees [28], score higher on the grade point average (GPA), are more productive in small group tutorials [36, 37] and achieve greater academic success through higher study effort [17]. The results of our study do not support the positive correlation of high levels of autonomous motivation and performance of NTS in students during simulation-based emergency training. Although all investigated students reported high levels of autonomous motivation, no impact on their performance of NTS was found.

One explanation for the results is that high levels of autonomous motivation might lead to deeper learning, more effort and consequently better scores in tests requiring factual knowledge [17, 38], but autonomous motivation might have no influence on performance which requires a transfer of knowledge to action [39].

Good performance of NTS requires indeed transferring effort (factual knowledge of skills has to be put in practice) and hence, our findings do not support the predictability of motivation regarding students` performance (NTS). Acquiring NTS is an ongoing process, which might be detached from the classical, factual learning approach- an approach which has been supported to have a positive correlation with motivation.

One strength of our study is the scrutinization of the correlation between motivation and performance of NTS in different year medical students, leading to more representative results than just investigating one cohort of undergraduates at one point of their medical studies. Hereby, possible time and study dependent alterations of motivation did not falsify our results.

Although the situational motivation within the investigated group of students was distributed heterogeneously, still no effect on performance of NTS was discovered.

The 2nd year students reported significantly higher levels of identified regulation and autonomous motivation, compared with the 3rd and 4th year students. The 1st year students showed higher levels of identified regulation than 4th year students. One possible explanation is that the 3rd and 4th year students might have reached a certain state of boredom and therefore do not identify themselves with the simulation trainings anymore. Boredom has shown to have a fading effect on intrinsic and identified regulation and thus autonomous motivation [40]. It can therefore be concluded that in order to prevent a decrease in autonomous motivation, repetitive exposure to SBET should be utilized advisedly.

One explanation for the fact that we did not detect a correlation between situational motivation and performance of NTS might be the partial homogeneity of our investigated group, regarding their levels of situational motivation. Further studies should analyze the effect of situational motivation on NTS, in different groups with different starting values of motivation.

We preferred the application of the SDT over other motivational theories, because the applicability and importance of SDT in medical education has been supported previously [19, 41]. The assessment of situational motivation, which is described in SDT, enables the comparison of different and repeated measurements [15]. That means one can measure the motivation to engage in a specific activity at an exact time point. Hereby, the motivational measures were narrowed to the emergency trainings to prevent biased results [33]. The reported levels of motivation were comparable in all investigated groups, which might lead to the assumption, that the SIMS was not able to assess adequately the motivational levels. But, as the SIMS has proven in several studies to be a reliable tool [26, 30], we believe that the investigated undergraduates had high levels of motivation and low levels would have been detected.

## Conclusion

In this study, high levels of autonomous situational motivation of students did not correlate with better performance of NTS in simulation-based emergency training. Therefore, enhancing medical students´ intrinsic motivation will not foster learning of NTS. Other teaching approaches need to be identified to improve performance of NTS.

### Limitations

Our prospective study was conducted in a cross-sectional design and therefore we did not analyze intra-individual variances.

## Data Availability

The datasets used and/or analysed during the current study are available from the corresponding author on reasonable request.

## Abbreviations

NTS: Non-technical skills
SDT: Self-determination theory
AS-NTS: Anaesthesiology students´ non-technical skills
SIMS: Situational motivation scale

## References

[1] Cooper JB, Newbower RS, Long CD, McPeek B. Preventable anesthesia mishaps: a study of human factors. Anesthesiology. 1978;49(6):399–406. 10.1097/00000542-197812000-00004 727541

[2] Gaba DM, Howard SK, Fish KJ, Smith BE, Sowb YA (2001). Simulation-based training in anesthesia crisis resource management (ACRM): a decade of experience. Simul Gaming.

[3] Fletcher G, Flin R, McGeorge P, Glavin R, Maran N, Patey R (2003). Anaesthetists' non-technical skills (ANTS): evaluation of a behavioural marker system. Br J Anaesth.

[4] Fletcher G, McGeorge P, Flin RH, Glavin RJ, Maran NJ (2002). The role of non-technical skills n anaesthesia: a review of current literature. Br J Anaesth.

[5] Flin R, Patey R. Improving patient safety through training in non-technical skills. BMJ; 2009;339:b3595. 10.1136/bmj.b3595. PMID: 19776108. 10.1136/bmj.b3595 19776108

[6] Donaldson MS, Corrigan JM, Kohn LT (2000). To err is human: building a safer health system: National Academies Press.

[7] Stewart GL, Barrick MR (2000). Team structure and performance: assessing the mediating role of intrateam process and the moderating role of task type. Acad Manag J.

[8] Weinger MB (2007). Experience≠ ExpertiseCan simulation be used to tell the difference?. J Am Soc Anesthesiol.

[9] Howard SK, Gaba DM, Fish KJ, Yang G, Sarnquist FH (1992). Anesthesia crisis resource management training: teaching anesthesiologists to handle critical incidents. Aviat Space Environ Med.

[10] Kiesewetter J, Gutmann J, Drossard S, Salas DG, Prodinger W, Mc Dermott F, et al. The Learning Objective Catalogue for Patient Safety in Undergraduate Medical Education–A Position Statement of the Committee for Patient Safety and Error Management of the German Association for Medical Education. GMS J Med Educ. 2016;33(1). 10.3205/zma001009. PMID: 26958647; PMCID: PMC4766934. 10.3205/zma001009 PMC4766934 26958647

[11] Zausig Y, Grube C, Boeker-Blum T, Busch C, Bayer Y, Sinner B (2009). Inefficacy of simulator-based training on anaesthesiologists' non-technical skills. Acta Anaesthesiol Scand.

[12] Yee B, Naik VN, Joo HS, Savoldelli GL, Chung DY, Houston PL (2005). Nontechnical skills in anesthesia crisis management with repeated exposure to simulation-based education. J Am Soc Anesthesiol.

[13] Wang CJ, Biddle SJ (2001). Young people’s motivational profiles in physical activity: a cluster analysis. J Sport Exercise Psychol.

[14] Vallerand RJ, Fortier MS, Guay F (1997). Self-determination and persistence in a real-life setting: toward a motivational model of high school dropout. J Pers Soc Psychol.

[15] Ratelle CF, Guay F, Vallerand RJ, Larose S, Senécal C (2007). Autonomous, controlled, and amotivated types of academic motivation: a person-oriented analysis. J Educ Psychol.

[16] Kusurkar R, Ten Cate TJ, Vos C, Westers P, Croiset G (2013). How motivation affects academic performance: a structural equation modelling analysis. Adv Health Sci Educ.

[17] Deci EL, Ryan RM (2008). Self-determination theory: a macrotheory of human motivation, development, and health. Can Psychol.

[18] Kusurkar R, Ten Cate TJ, Van Asperen M, Croiset G (2011). Motivation as an independent and a dependent variable in medical education: a review of the literature. Medical Teacher..

[19] ten Cate OTJ, Kusurkar RA, Williams GC (2011). How self-determination theory can assist our understanding of the teaching and learning processes in medical education. AMEE guide no. 59. Medical Teacher.

[20] Williams GC, Saizow RB, Ryan RM (1999). The importance of self—determination theory for. Acad Med.

[21] Deci E, Ryan RM (1985). Intrinsic motivation and self-determination in human behavior: Springer Science & Business Media.

[22] Ryan RM, Deci EL (2000). Self-determination theory and the facilitation of intrinsic motivation, social development, and well-being. Am Psychol.

[23] Ntoumanis N (2002). Motivational clusters in a sample of British physical education classes. Psychol Sport Exerc.

[24] Deci EL, Eghrari H, Patrick BC, Leone DR (1994). Facilitating internalization: the self-determination theory perspective. J Pers.

[25] Koestner R, Losier GF (2002). Distinguishing three ways of being highly motivated: a closer look at introjection, identification, and intrinsic motivation.

[26] Deci EL, Ryan RM (2010). Intrinsic motivation. The corsini encyclopedia of psychology.

[27] Sobral DT (2004). What kind of motivation drives medical students' learning quests?. Med Educ.

[28] Guay F, Vallerand RJ, Blanchard C (2000). On the assessment of situational intrinsic and extrinsic motivation: the situational motivation scale (SIMS). Motiv Emot.

[29] Moll-Khosrawi P, Kamphausen A, Hampe W, Schulte-Uentrop L, Zimmermann S, Kubitz JC (2019). Anaesthesiology students’ non-technical skills: development and evaluation of a behavioural marker system for students (AS-NTS). BMC Med Educ.

[30] Knörzer L, Brünken R, Park B (2016). Facilitators or suppressors: effects of experimentally induced emotions on multimedia learning. Learn Instr.

[31] Gillet N, Vallerand RJ, Lafreniere M-AK, Bureau JS (2013). The mediating role of positive and negative affect in the situational motivation-performance relationship. Motiv Emot.

[32] McClelland DC (1985). How motives, skills, and values determine what people do. Am Psychol.

[33] Standage M, Gillison F, Treasure DC (2007). Self-determination and motivation in physical education.

[34] Cohen J (2013). Statistical power analysis for the behavioral sciences: Routledge.

[35] Dolmans DH, Wolfhagen IH, VAN DER VLEUTEN CP (1998). Thinking about student thinking: motivational and cognitive processes influencing tutorial groups. Acad Med.

[36] Das Carlo M, Swadi H, Mpofu D (2003). Medical student perceptions of factors affecting productivity of problem-based learning tutorial groups: does culture influence the outcome?. Teach Learning Med.

[37] Webb CT, Sedlacek W, Cohen D, Shields P, Gracely E, Hawkins M (1997). The impact of nonacademic variables on performance at two medical schools. J Natl Med Assoc.

[38] De Bruin AB, Van De Wiel MW, Rikers RM, Schmidt HG (2005). Examining the stability of experts’ clinical case processing: an experimental manipulation. Instr Sci.

[39] Pelaccia T, Viau R (2017). Motivation in medical education. Medical Teacher..

[40] Kusurkar R, Croiset G, Custers E, Ten Cate O. Development of motivation theories and how they relate to development of medical education. Motivation Med Students. 2012;37. 10.1097/ACM.0b013e318253cc0e 22534597

[41] Harter S (1981). A new self-report scale of intrinsic versus extrinsic orientation in the classroom: motivational and informational components. Dev Psychol.

